# Supplementary material for: Orphan GPR26 Counteracts Early Phases of Hyperglycemia-Mediated Monocyte Activation and Is Suppressed in Diabetic Patients
Source: Biomedicines. 2022 Jul 19;10(7):1736. doi: 10.3390/biomedicines10071736 (PMC9312814; doi:10.3390/biomedicines10071736)
Supplement: Supplementary file 1 [file biomedicines-10-01736-s001.zip › biomedicines-1787030-table S1S2.pdf]

Supplementary material and methods

Supplementary Table S1. Sequence of primers against the human genes analyzed by qPCR.

Gene	Forward primer	Reverse primer
GPR26	5'- GGGTGGGACATCTGTGTGTT -3'	5'- ACGGGCACAGACTTATGC -3'
B2M	5'- GATGAGTATGCCTGCCGTGT -3'	5'- TGCGGCATCTTCAAACCTCC -3'

Supplementary Table S2. List of antibodies for western blot (WB)

**Antigen**	**supplier**	**Clone / Cat. no.**	**Concentration**
**Primary antibodies for WB**			
Caspase-3	CST	8G10 / 9665	1:1000
Cleaved caspase-3	CST	5A1E / 9664	1:1000
LC3B	CST	D11 / 3868	1:1000
p38 MAPK	CST	9212	1:1000
Phospho-p38 MAPK(p-p38)	CST	9211	1:1000
p44/42 MAPK (Erk1/2)	CST	9102	1:1000
phospho-p44/42 MAPK (Erk1/2) (Thr202/Tyr204)	CST	9101	1:1000
SQSTM1/p62	CST	5114	1:1000
NF-κB (p65)	CST		1:1000
β-actin	SCB	2418	1:4000
**Secondary antibody for WB**			
Goat anti -rabbit IgG (HRP)	CST	7074	1:2000

CST: Cell Signaling Technology; SCB: Santa Cruz Biotechnology
